# Evaluation of profibrotic gene transcription in renal tissues from cats with naturally occurring chronic kidney disease

**DOI:** 10.1111/jvim.15801

**Published:** 2020-05-29

**Authors:** Bianca N. Lourenço, Amanda E. Coleman, Jaime L. Tarigo, Roy D. Berghaus, Cathy A. Brown, Daniel R. Rissi, James B. Stanton, Scott A. Brown, Chad W. Schmiedt

**Affiliations:** ^1^ Department of Small Animal Medicine and Surgery College of Veterinary Medicine, University of Georgia Athens Georgia USA; ^2^ Department of Pathology College of Veterinary Medicine, University of Georgia Athens Georgia USA; ^3^ Department of Population Health College of Veterinary Medicine, University of Georgia Athens Georgia USA; ^4^ Departments of Small Animal Medicine & Surgery and Physiology & Pharmacology College of Veterinary Medicine, University of Georgia Athens Georgia USA

**Keywords:** feline, hypoxia‐inducible factor, matrix metalloproteinases, tissue inhibitor of metalloproteinase, transforming growth factor, tubulointerstitial fibrosis, vascular endothelial growth factor

## Abstract

**Background:**

Increased gene transcription of hypoxia‐induced mediators of fibrosis in renal tissue has been identified in experimentally induced, ischemic chronic kidney disease (CKD).

**Objective:**

To characterize hypoxia‐induced profibrotic pathways in naturally occurring CKD in cats.

**Animals:**

Twelve client‐owned cats with CKD and 8 healthy control cats.

**Methods:**

In this prospective, cross‐sectional study, bilateral renal tissue samples were assessed histologically for inflammation, tubular atrophy, and fibrosis, and by reverse transcription‐quantitative PCR for characterization of transcript levels of hypoxia‐inducible factor‐1α (*HIF1A*), matrix metalloproteinases‐2 (*MMP2*), ‐7 (*MMP7*), and ‐9 (*MMP9*), tissue inhibitor of metalloproteinase‐1 (TIMP1), transforming growth factor‐β1 (*TGFB1*), and vascular endothelial growth factor‐A (*VEGFA*). Linear mixed models were used to compare gene transcription between diseased and healthy kidneys, and to examine the association between transcript levels and serum creatinine concentration for all cats, and between transcript levels and histologic scores of diseased kidneys.

**Results:**

Kidneys from cats with CKD had significantly higher transcript levels of *HIF1A*, *MMP2*, *MMP7*, *MMP9*, *TIMP1*, and *TGFB1* (all *P* < .001), and lower levels of *VEGFA* (*P* = .006) than those from control cats. Transcript levels of *MMP7* (*P* = .05) and *TIMP1* (*P* = .005) were positively associated with serum creatinine in cats with CKD, but not in control cats. In diseased kidneys, transcript levels of *MMP2* (*P* = .002), *MMP7* (*P* = .02), and *TIMP1* (*P* = .02) were positively, whereas those of *VEGFA* (*P* = .003) were negatively, associated with histologic score severity.

**Conclusion and Clinical Significance:**

Evaluation of the expression of the corresponding proteins in larger populations could identify therapeutic targets and/or biomarkers of tubulointerstitial fibrosis in cats.

**Abbreviations**
CKD: chronic kidney disease
*GAPDH*: gene for glyceraldehyde 3‐phosphate dehydrogenase
*HIF1A*: gene for hypoxia‐inducible factor‐1α
HIF‐1α: hypoxia‐inducible factor‐1α
MMP: matrix metalloproteinase
*MMP2*: gene for matrix metalloproteinase‐2
*MMP7*: gene for matrix metalloproteinase‐7
*MMP9*: gene for matrix metalloproteinase‐9
*RPS7*: gene for ribosomal protein S7
sCr: serum creatinine concentration
SDMA: serum symmetric dimethylarginine concentration
SUN: serum urea nitrogen
TGF: transforming growth factor
*TGFB1*: gene for transforming growth factor‐β1
TIMP: tissue inhibitor of metalloproteinase
*TIMP1*: gene for tissue inhibitor of metalloproteinase‐1
UPC: urinary protein‐to‐creatinine ratio
USG: urine specific gravity
VEGF: vascular endothelial growth factor
*VEGFA*: gene for vascular endothelial growth factor‐A

## 1 INTRODUCTION

Tubulointerstitial fibrosis, a key histologic feature of chronic kidney disease (CKD) in cats,^1^ is correlated with the degree of functional renal impairment.^2^, ^3^ Across mammalian species, this pattern of histologic change is commonly observed in the advanced and end‐stages of CKD.^4^, ^5^ However, in cats, renal fibrosis is present from the early stages of disease.^1^, ^3^

Growing evidence has emphasized the critical role of tubulointerstitial hypoxia in the development and progression of renal fibrosis and CKD.^6^, ^7^, ^8^ Although the processes leading to fibrosis can initially promote normal tissue repair in response to renal injury,^9^ the development of fibrosis has been proposed as a maladaptive response. Fibrosis can alter renal microcirculation and increase the distance for oxygen diffusion between renal tubules and capillaries, thereby promoting chronic renal hypoxia.^7^, ^10^ On a molecular level, the cellular and tissular responses to hypoxia are orchestrated by the complex interaction of cytokines and transcription factors. Accumulation of hypoxia‐inducible factor (HIF)‐1α in hypoxic regions initiates hypoxia‐adaptive responses,^8^ and is a key driving force for the progression of CKD in rodents.^11^ Vascular endothelial growth factor (VEGF)‐A, a potent proangiogenic factor, is stimulated by renal hypoxia via HIF‐1α.^12^, ^13^ Both underexpression and overexpression of VEGF‐A have been associated with worsening of kidney disease, demonstrating this cytokine's complex pathophysiologic role.^14^ Other fibrogenic factors that have been suggested as central to the development of renal fibrosis under hypoxic conditions include transforming growth factor (TGF)‐β1,^7^, ^15^ the matrix metalloproteinases (MMPs), particularly MMP‐2, ‐7, and ‐9, and their tissue inhibitors (TIMPs).^16^, ^17^

Although alterations in urinary TGF‐β1 and VEGF‐A occur in cats with CKD,^18^, ^19^, ^20^, ^21^ the roles of HIF‐1α and MMPs remain to be characterized in spontaneous renal disease in cats. There is increased transcription of the genes for MMP‐2 (*MMP2*), MMP‐7 (*MMP7*), MMP‐9 (*MMP9*), TIMP‐1 (*TIMP1*), and TGF‐β1 (*TGFB1*), and decreased transcription of the gene for VEGF‐A (*VEGFA*), in the kidneys of cats subjected to unilateral, transient renal ischemia, relative to those of healthy controls.^22^ In that study, although there was no difference in transcript abundance of *HIF1A* between groups, renal transcript levels of *HIF1A*, *MMP2*, *MMP7*, and *TIMP1* were positively and strongly correlated with worsening degrees of fibrosis in kidneys exposed to transient ischemia.

The objective of the present study was to characterize the renal transcription of hypoxia‐induced profibrotic pathways in naturally occurring CKD in cats. It was hypothesized that as in ischemia‐induced experimental CKD, and compared to tissues from healthy control cats, gene transcript levels of *HIF1A*, *MMP2*, *MMP7*, *MMP9*, *TIMP1*, and *TGFB1* would be increased, and those of *VEGFA* would be decreased, in renal tissues from cats with naturally occurring CKD. A secondary objective was to examine the association between profibrotic gene transcription and histologic renal lesions. It was hypothesized that transcript levels of the profibrotic mediators would be positively, whereas those of the proangiogenic factor *VEGFA* would be negatively, associated with severity of histologic changes in affected kidneys.

## 2 MATERIALS AND METHODS

### 2.1 Study design

This was a prospective, cross‐sectional study performed on renal tissue samples obtained from client‐owned cats diagnosed with naturally occurring CKD (CKD group) and from healthy control cats. The University of Georgia Institutional Animal Care and Use Committee approved all activities related to this study (Animal Use Protocol A2017 05‐008‐Y3‐A1).

### 2.2 Animals

Renal tissue samples from cats of the CKD group were obtained immediately postmortem from cats that presented for euthanasia, or within 1 hour of witnessed death in those dying of natural causes, at primary care and referral veterinary hospitals in the states of Georgia and North Carolina. Cats were considered for enrollment if they had documented or suspected CKD, based on the presence of 1 or more of the following: urine specific gravity (USG) <1.035 absent an identifiable extrarenal cause, azotemia (ie, serum creatinine concentration [sCr] ≥1.6 mg/dL), serum symmetric dimethylarginine concentration (SDMA) >14 μg/dL, or renal proteinuria (ie, urinary protein‐to‐creatinine ratio [UPC] >0.4). Cats were included if renal histology, performed by 1 of 2 veterinary pathologists (C. A. B. or D. R. R.), revealed chronic lesions (ie, glomerulosclerosis, tubular atrophy, tubulointerstitial fibrosis, or any combination of these) consistent with CKD. Cats may have been euthanized or died of natural causes related to renal or extrarenal disease. Cats were excluded if they received a renin‐angiotensin‐aldosterone system antagonist (ie, an angiotensin‐converting enzyme inhibitor, angiotensin receptor blocker, or mineralocorticoid receptor antagonist) or a short‐acting corticosteroid in the 14 days preceding euthanasia or natural death, or if they received a depot corticosteroid injection in the 6 months before euthanasia or natural death. Cats were also excluded if they were affected by uncontrolled hyperthyroidism (total T4 > upper limit of laboratory reference range at most recent sampling) or congestive heart failure. All owners were required to read and sign a form consenting to their pet's participation in the study.

Samples from the control group were collected from adult cats that were euthanized as part of population control measures at a local animal control facility, and from adult purpose‐bred research cats participating in unrelated terminal studies having no impact on renal structure and/or function. These cats were considered to be healthy based on normal findings of physical exam and necropsy, and were deemed to have normal renal function and structure on the basis of renal histology, serum biochemistry, and urine analyses (ie, sCr <1.6 mg/dL, SDMA ≤14 μg/dL, USG >1.035, and UPC <0.4). For healthy intact cats, an UPC < 0.6 was considered acceptable for inclusion, provided all other renal function biomarkers (ie, sCr, serum urea nitrogen [SUN], SDMA, and USG) and renal histology were normal.^23^ Cats recruited from the animal control facility population were additionally screened for retroviral infections using a combined feline immunodeficiency virus antibody and feline leukemia virus antigen test (SNAP FIV/FeLV Combo Test, IDEXX Laboratories, Westbrook, Maine), and were excluded if positive for either.

### 2.3 Blood and urine sample collection and processing

For control cats identified at the animal control facility, blood and urine samples were collected via cardiocentesis and cystocentesis, respectively, immediately after euthanasia with pentobarbital. For purpose‐bred control cats, blood and urine samples were collected via jugular venipuncture and cystocentesis, respectively, after sedation with intramuscular buprenorphine (0.03 mg/kg), acepromazine (0.1 mg/kg), and midazolam (0.3 mg/kg), immediately before euthanasia with pentobarbital. Serum, ethylenediaminetetraacetic acid‐anticoagulated blood, and urine samples were submitted for analysis within 1 hour of collection.

Client‐owned cats enrolled in the CKD group did not undergo blood and urine sampling. Information pertaining to each cat's renal function was obtained from review of the individual's medical record.

### 2.4 Clinical laboratory analyses

Complete blood count and serum biochemistry profile including SDMA, urinalysis, and UPC measurement were performed for each control cat. Serum SDMA was measured by an external laboratory (IDEXX Laboratories). All other clinical laboratory analyses were performed by the Clinical Pathology Laboratory of the University of Georgia.

### 2.5 Renal tissue sample collection and processing

For all cats, renal tissue samples were collected within 1 hour of death or euthanasia by 1 of the investigators or a trained individual. For each cat, both kidneys were removed through a midline laparotomy and sectioned longitudinally. To maintain equal proportions of renal cortex, medulla, and corticomedullary junction, one‐half of each kidney was minced and placed separately in RNA stabilization solution (RNAlater stabilization solution, QIAGEN, Valencia, California), and the remaining portion was placed in neutral‐buffered 10% formalin. After overnight incubation at 4°C, tissues were removed from RNA stabilization solution, homogenized with a mortar and pestle, divided into 30 mg aliquots, and stored at −80°C until further analysis.

### 2.6 Gene transcription analysis

Reverse transcription and quantitative PCR were performed as previously described.^22^ For each sample, total RNA was extracted from 30 mg of tissue homogenate using a commercially available RNA extraction kit (RNeasy Plus Mini Kit, QIAGEN). The integrity and purity of the isolated RNA was confirmed by quantification using a spectrophotometer (NanoDrop Spectrophotometer, Thermo Fisher Scientific, Waltham, Massachusetts), followed by the visualization of 18S and 28S ribosomal bands on 1.2% agarose gels. A 260/280 ratio of >1.80 was used as evidence of RNA purity.^24^ The average 260/280 ratio for the samples evaluated was 2.06 ± 0.06. A total of 1 μg of RNA extracted from each sample was treated with deoxyribonuclease (ezDNase, Invitrogen, Carlsbad, California), and reverse‐transcribed using a cDNA reaction master mix (SuperScript IV VILO Master Mix, Invitrogen). Quantification was performed in an automated thermocycler (CFX96, Bio‐Rad Laboratories, Hercules, California) using 20 μL reactions containing 10 μL of SYBR Green Supermix (SsoAdvanced SYBR Green Supermix, Bio‐Rad Laboratories), 5 pmol of each primer (Table 1), and 9 μL of complementary DNA sample at a 1:40 dilution. Thermal cycling conditions consisted of an activation step at 95°C for 30 seconds, followed by 36 amplification cycles (95°C for 15 seconds for denaturation and 60°C for 30 seconds for annealing and extension), and a melt curve step (60°C‐95°C, increasing at increments of 0.5°C every 5 seconds). All reactions were performed in triplicate and average values were used for further analyses. A no reverse transcriptase control was included for each sample and 3 no template controls were used in each plate. In accordance with the sample maximization method,^25^ for each gene, all samples were analyzed concurrently, divided in 2 96‐well plates. A standard sample was analyzed in triplicate with glyceraldehyde 3‐phosphate dehydrogenase (*GAPDH*) primers in each plate and used as an interrun control for the entire gene study.

**TABLE 1:** Primer sequences for quantitative polymerase chain reaction

Gene	Ensemble ID/ NCBI access no.	Primer sequence (5′ → 3′)	Fragment size (bp)	Source
*GAPDH*	ENSFCAG 00000006874	For: GCTGCCCAGAACATCATCC rev: GTCAGATCCACGACGGACAC	134	Riedel et al^27^
*RPS7*	NM_001009832	For: GTCCCAGAAGCCGCACTT T rev: CACAATCTCGCTCGGGAA AA	74	Kessler et al^28^
*HIF1A*	XM_001493206	For: TTGGCAGCAATGACACAGACACTG rev: TTGAGTGCAGGGTCAGCACTACTT	175	Agaoglu et al^29^
*MMP2*	XM_003998042.2	For: AGACAAGTTCTGGAGGTACAATG rev: CGCCCTTGAAGAAGTAGCTGT	149	Lourenço et al^22^
*MMP7*	XM_003992303.2	For: CTTTGCAAGAGGAGCTCACG rev: AATTCCTAGACCCCTGCCGT	148	Lourenço et al^22^
*MMP9*	XM_003983412.4	For: GCTTCTGGAGGTTCGACGTG rev: CAATAGAAGCGGTCCTGGCA	148	Lourenço et al^22^
*TGFB1*	AY425617.1	For: AGCACGTGGAGCTGTACCAGAAAT rev: TCCAGTGACATCAAAGGACAGCCA	110	Agaoglu et al^30^
*TIMP1*	XM_011291721.2	For: TCTCATCGCCGGAAAACTGC rev: AGCCAGCAGCATAGGTCTTG	122	Lourenço et al^22^
*VEGFA*	AB071947.1	For: TTTCTGCTCTCTTGGGTGCATTGG rev: TGCGCTGGTAGACATCCATGAACT	139	Agaoglu et al^29^

Abbreviations: For: forward primer; *GAPDH*, gene for glyceraldehyde 3‐phosphate dehydrogenase; *HIF1A*, gene for hypoxia‐inducible factor‐1α; *MMP2*, gene for matrix metalloproteinase‐2; *MMP7*, gene for matrix metalloproteinase‐7; *MMP9*, gene for matrix metalloproteinase‐9; NCBI, National Center for Biotechnology Information; rev: reverse primer; *RPS7*, gene for ribosomal protein S7; *TGFB1*, gene for transforming growth factor‐β1; *TIMP1*, gene for tissue inhibitor of metalloproteinase‐1; *VEGFA*, gene for vascular endothelial growth factor‐A.

Transcript levels of the target genes *HIF1A*, *MMP2*, *MMP7*, *MMP9*, *TGFB1*, *TIMP1*, and *VEGFA* were normalized to those of 2 reference genes, *GAPDH* and ribosomal protein S7 (*RPS7*), by using commercially available software (CFX Manager, Bio‐Rad Laboratories) that utilizes the geNorm method.^26^ Reference genes were selected based on the efficiency of their primers, and their stability value (M value <0.5) and coefficient of variance (<0.25) when tested in a complete set of the experiment's samples. Both reference genes, *GAPDH* and *RPS7*, had a stability value and coefficient of variation of 0.35 and 0.12, respectively. After normalization, for each target gene, transcript levels were scaled to those of the lowest sample.

Gene specific primers were selected from previously reported studies (Table 1).^22^, ^27^, ^28^, ^29^, ^30^ Primers were previously validated for reverse transcription‐quantitative PCR analysis in feline renal tissue homogentes.^22^

### 2.7 Gross and histologic renal evaluation

Gross renal evaluation was performed by the study investigator collecting the samples (B. N. L. or A. E. C.). When specimens were collected by a trained individual other than an investigator, gross evaluation was carried out in formalin‐fixed samples by a board‐certified veterinary pathologist (C. A. B. or D. R. R.). Three‐micrometer‐thick sections of formalin‐fixed, paraffin‐embedded tissues were stained with Masson's trichrome, hematoxylin and eosin, and periodic acid‐Schiff and hematoxylin. For each kidney, 10 consecutive ×20 fields of cortex and corticomedullary junction were scored for the degree of fibrosis, inflammation, and tubular atrophy. A consensus score was arrived at by conference of 2 board‐certified veterinary pathologists (C. A. B. and D. R. R.), as previously described.^22^, ^31^ Briefly, the degree of fibrosis was evaluated in Masson's trichrome‐stained sections and scored as 0 (absent; no increase compared to normal), 1 (mild, rare foci/segments of fibrosis involving <20% of the examined field), 2 (moderate, fibrotic segments involving 20%‐30% of the examined field), or 3 (severe, fibrotic segments involving >30% of the examined field). Inflammation (ie, presence of lymphocytes, plasma cells, macrophages, or some combination of these) was evaluated in hematoxylin and eosin‐stained slides and scored as 0 (absent, no inflammatory cells), 1 (mild, <10% of the examined field affected), 2 (moderate, 10%‐50% of the examined field affected), or 3 (severe, >50% of the examined field affected). Lastly, tubular atrophy was scored in periodic acid‐Schiff‐ and hematoxylin‐stained sections as 0 (no atrophy), 1 (mild, fewer than 10 scattered atrophic tubules per field), 2 (moderate, linear streaks of tubular atrophy often with fibrosis and inflammation), or 3 (severe, 2 or more streaks of tubular atrophy per field). Numeric scores of the 20 (10 cortical and 10 corticomedullary) examined fields were used to calculate median scores for inflammation, tubular atrophy, and fibrosis for each kidney. Median scores that fell between 2 classification categories were rounded to the nearest integer.

### 2.8 Statistical analysis

Statistical analyses were performed by using commercially available software packages (Stata 16.0 for Windows, Stata Corporation, College Station, Texas, and GraphPad Prism for Mac, version 7, GraphPad Software Inc, La Jolla, California). A significance threshold of .05 was used.

The distribution of continuous demographic and clinical data by disease group was examined for normality by visual assessment of histograms and normal quantile plot, and by the Shapiro‐Wilk test. Normally distributed data are presented as mean ± SD and compared between groups using the 2‐sample, unequal variance *t* test. Nonnormally distributed data are presented as median (range) and compared using the Mann‐Whitney *U* test. For purposes of comparison between disease groups, USG values reported as >1.060 and SUN values reported as >100 or >180 mg/dL were assigned a value of 1.061 and 101 or 181, respectively. Categorical data were compared by using Fisher's exact test.

Scaled, normalized gene transcript levels linear mixed model residuals were evaluated for the assumption of normality by assessment of histograms and Q‐Q plots. Gene transcript levels underwent a natural logarithmic transformation, improving the normality of the residuals. Linear mixed models were used to compare natural log‐transformed, scaled, normalized gene transcript levels of each target gene between groups. The full model for each gene transcript variable included a fixed factor for group and random factor of cat to account for within‐cat correlation, as samples from the right and left kidneys were evaluated from each cat. Alternative models also included a fixed covariate of age to adjust for effects of age, or sCr and a sCr by group interaction to test for association of sCr and gene transcript levels. Cats for which clinicopathologic data were obtained more than 91 days before euthanasia or natural death and renal sampling were excluded from this analysis.

For kidneys from cats with CKD, the association between ordinal histologic lesion scores and the transcript levels of each target gene was evaluated using linear mixed models with a fixed covariate of histologic score. The linearity of ordinal histologic scores was evaluated by including both a linear term and k − 2 indicator variables, where k is the number of score categories. If the inclusion of the indicator variables did not significantly improve model fit, histologic score was treated as a continuous predictor. Satterthwaite degrees of freedom method and restricted maximum likelihood estimation were used. Equality of group variances was not assumed for any of the analyses.

## 3 RESULTS

### 3.1 Animals

Samples from 13 cats with a diagnosis of CKD and from 12 control cats (5 purpose‐bred and 7 feral cats) were collected between July 2017 and March 2019. Samples from 4 control cats and 1 CKD cat that did not meet the enrollment criteria were excluded from analysis. Tissues from 1 purpose‐bred control cat were excluded because of unexplained, marked right hind limb edema. Reasons for exclusion of 3 feral control cats included a concurrent diagnosis of pyometra (n = 1), positive feline immunodeficiency virus antibody test (n = 1), and concurrent leukocytosis and bacteriuria (n = 1). One CKD cat was excluded because of having received a depot corticosteroid injection 10 days before euthanasia. Twelve cats with CKD and 8 healthy control cats met the enrollment criteria and were included in the present study; all included cats underwent euthanasia.

A summary of demographic and clinical data of included cats is presented in Table 2. Although the ages of 4 feral control cats were estimated, cats of the CKD group were significantly older than healthy controls. There was a significant difference in sex and neuter status between groups, with the control group having a greater proportion of sexually intact cats. There were no significant differences in body weight or breed distribution between groups. Consistent with the inclusion criteria and definitions used to designate cats as affected by CKD, significant differences in SUN, sCr, USG, and UPC were observed between groups. All neutered cats included in the control group had a UPC ≤0.19.

**TABLE 2:** Clinical and clinicopathologic data from cats with chronic kidney disease (n = 12) and from healthy control cats (n = 8). Numerical data are presented as mean ± SD or median (range), where appropriate. Number of cats for which data are available is provided if different from the number of individuals in each group

	Chronic kidney disease group	Healthy control group	*P* value
Number included	12	8	
Age (years)	17.4 (3.1‐19.4)	1.7 (1‐7)	<.001
Sex			.005
Female spayed	7	0	
Female intact	0	3	
Male neutered	5	4	
Male intact	0	1	
Breed			.24
Domestic short haired	8	8	
Domestic medium haired	3	0	
Abyssinian	1	0	
Body weight (kg)	3.77 ± 1.42	4.22 ± 0.95	.44
n	10	8	
Serum creatinine concentration (mg/dL)	5.7 ± 3.2	0.9 ± 0.5	<.001
Serum urea nitrogen concentration (mg/dL)	92.0 ± 41.9	24.9 ± 5.1	<.001
Serum symmetric dimethylarginine (μg/dL)	14.5 (9‐20)	10.5 (6‐14)	.60
n	2	8	
Urine specific gravity	1.016 ± 0.003	1.051 ± 0.007	<.001
n	8	8	
Urinary protein‐to‐creatinine ratio	0.70 (0.39‐23.41)	0.19 (0.07‐0.41)	.02
n	3	8	
Days elapsed between last clinicopathologic data and euthanasia	11.5 (0‐525)	0 (0‐0)	.01
IRIS CKD stage^a^		N/A	
1	1		
2	2		
3	2		
4	7		

Abbreviations: CKD, chronic kidney disease; IRIS, International Renal Interest Society.

a IRIS CKD stage assumes that the most recent serum creatinine concentration measurement represented the cat’s stable value.

All cats in the CKD group were euthanized for clinical signs presumed to be related to kidney disease, including persistent lethargy, inappetence, chronic weight loss, or some combination of these. Acute‐on‐CKD was considered likely for 2 of these cats. As biochemical data from client‐owned cats were not acquired during the same hospital visit as euthanasia in all instances, but rather obtained via review of medical record, the median (range) time between the date of last creatinine measurement and euthanasia was longer for CKD [11.5 (0‐525) days] than for control [0 (0‐0) days] cats. The time between collection of clinicopathologic data and euthanasia was greater than 91 days for only 1 cat. When the most recent sCr measurement was used to stage cats of the CKD group using categories defined by the International Renal Interest Society,^32^ the majority of cats were affected by advanced CKD stages (Table 2). Systolic systemic arterial blood pressure and UPC measurements were not consistently available for complete CKD substaging.

Concurrent diseases/conditions were diagnosed in 5 cats from the CKD group and included myocardial hypertrophy (n = 1), pancreatic Langerhans cell histiocytosis, extensive necrotizing enteritis, and mild myocardial hypertrophy (n = 1), possible *Streptococcus* sp. (alpha) urinary tract infection (growth from subculture of enrichment broth—uncertain clinical relevance) undergoing antibiotic treatment for 13 days before euthanasia (n = 1), bilateral nephrolithiasis with left ureteral obstruction treated with ureterotomy 16 days before euthanasia (n = 1), and suspect middle ear neoplasm (n = 1).

Medications prescribed in the 6 weeks preceding euthanasia included mirtazapine (n = 2), amoxicillin/clavulanic acid (n = 2), atenolol (n = 1), chitosan and calcium carbonate (n = 1), aluminum hydroxide (n = 1), ranitidine (n = 1), sucralfate (n = 1), and lactulose (n = 1). Subcutaneous fluids were being administered regularly by the owner in 2 cases.

Six cats in the CKD group were fed a commercially available prescription renal diet (Hill's k/d in 5 cases, of which 1 was also receiving Hill's g/d, and Royal Canin Feline Renal Support D in the remaining case). One cat was fed a maintenance diet (Pro Plan Focus urinary tract formula). Dietary information was not available for 5 CKD cats and none of the feral cats. Purpose‐bred control cats were fed an adult maintenance diet (cat chow).

### 3.2 Gene transcription analyses

**FIGURE 1:**
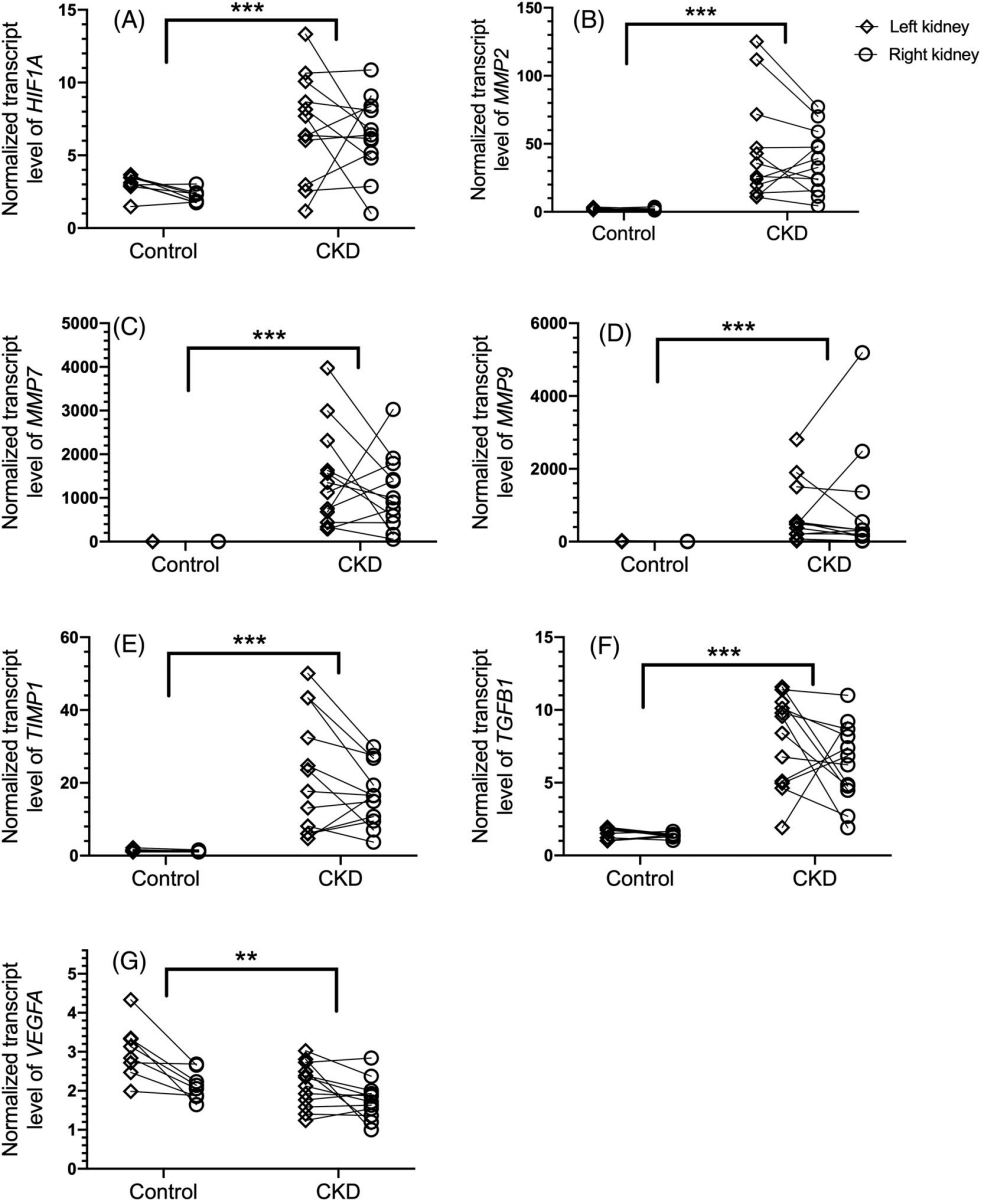
Dot plots of normalized gene transcript levels of *HIF1A* (A), *MMP2* (B), *MMP7* (C), *MMP9* (D), *TIMP1* (E), *TGFB1* (F), and *VEGFA* (G) in renal tissue homogenates from each kidney of cats with chronic kidney disease (n = 12) and healthy control cats (n = 8). Levels of each target gene were normalized to those of the reference genes *GAPDH* and *RPS7*. For each gene, transcript levels are scaled to those of the lowest sample; note, therefore, that scales differ among genes. Values from the left (open diamond) and right kidney (open circle) of the same individual are connected by solid lines. **P < .01; ***P < .001. CKD, chronic kidney disease; *HIF1A*, gene for hypoxia‐inducible factor‐1α; MMP2, gene for matrix metalloproteinase‐2; *MMP7*, gene for matrix metalloproteinase‐7; *MMP9*, gene for matrix metalloproteinase‐9; *TGFB1*, gene for transforming growth factor‐β1; *TIMP1*, gene for tissue inhibitor of metalloproteinase‐1; *VEGFA*, gene for vascular endothelial growth factor‐A

Renal tissue samples from cats of the CKD group had significantly higher transcript levels of *HIF1A*, *MMP2*, *MMP7*, *MMP9*, *TIMP1*, and *TGFB1* (all *P* < .001), and lower levels of *VEGFA* (*P* = .006) than those of the control group (Figure 1).

Age was not significantly associated with transcript levels of any of the 7 genes evaluated (*P* > .30). Differences in transcript levels of all genes remained statistically significant when adjusted for the difference in age between the cats in each group (*P* ≤ .035 for all genes).

### 3.3 Association between gene transcript levels and serum creatinine concentration

For healthy control cats, there was no significant association between sCr and transcript level of any gene evaluated. Conversely, for cats with CKD, there was a significant, positive association between sCr and renal tissue transcript levels of *MMP7* and *TIMP1*. Data from the cat for which the most recent sCr measurement were obtained 525 days before euthanasia was excluded from this analysis. Slopes (unit change in ln‐transformed transcript levels per 1 mg/dL change in creatinine) and *P* values from a linear mixed model testing these associations are presented in Table 3.

**TABLE 3:** Association between serum creatinine concentration (mg/dL) and natural log‐transformed bilateral renal tissue homogenate gene transcript levels in healthy control cats (n = 8) and cats with chronic kidney disease (n = 11) using a linear mixed model

Gene	Group	Slope (SE)	*P* value
*HIF1A*	Control	−0.21 (0.15)	.21
	CKD	−0.01 (0.05)	.86
*MMP2*	Control	0.49 (0.40)	.25
	CKD	0.12 (0.06)	.06
*MMP7*	Control	0.53 (0.32)	.14
	CKD	0.14 (0.06)	.05
*MMP9*	Control	1.14 (0.85)	.20
	CKD	0.15 (0.11)	.20
*TGFB1*	Control	0.19 (0.11)	.12
	CKD	−0.02 (0.04)	.65
*TIMP1*	Control	0.04 (0.17)	.81
	CKD	0.13 (0.04)	.005
*VEGFA*	Control	−0.01 (0.14)	.95
	CKD	−0.04 (0.02)	.07

*Notes*. Linear mixed model with fixed effect = group, creatinine, group*creatinine; random effect = cat. Slopes represent the change in ln(transcript levels) corresponding to a 1 mg/dL increase in serum creatinine. One cat in the chronic kidney disease group did not have a serum creatinine measurement within 91 days of euthanasia and was not included in the analysis.

Abbreviations: *HIF1A*, gene for hypoxia‐inducible factor‐1α; *MMP2*, gene for matrix metalloproteinase‐2; *MMP7*, gene for matrix metalloproteinase‐7; *MMP9*, gene for matrix metalloproteinase‐9; *RPS7*, gene for ribosomal protein S7; *TGFB1*, gene for transforming growth factor‐β1; *TIMP1*, gene for tissue inhibitor of metalloproteinase‐1; *VEGFA*, gene for vascular endothelial growth factor‐A.

### 3.4 Gross and histologic renal evaluation

Marked asymmetry in renal size and morphology was present in 3 CKD cats (ie, “big kidney‐little kidney” syndrome). In each, the right kidney was abnormally large, whereas the left kidney was abnormally small.

Histologically, cats with CKD were diagnosed with typical ischemic CKD (ie, chronic tubular atrophy and tubulorrhexis with interstitial inflammation, lipid, and fibrosis; n = 7), ischemic CKD with oxalosis (n = 2), amyloidosis (n = 1), membranoproliferalive glomerulonephritis (n = 1), and possible renal maldevelopment with secondary ischemic changes (n = 1). Urothelial proliferative changes were noted in the papilla and pelvis of 7 cats with CKD.

**FIGURE 2:**
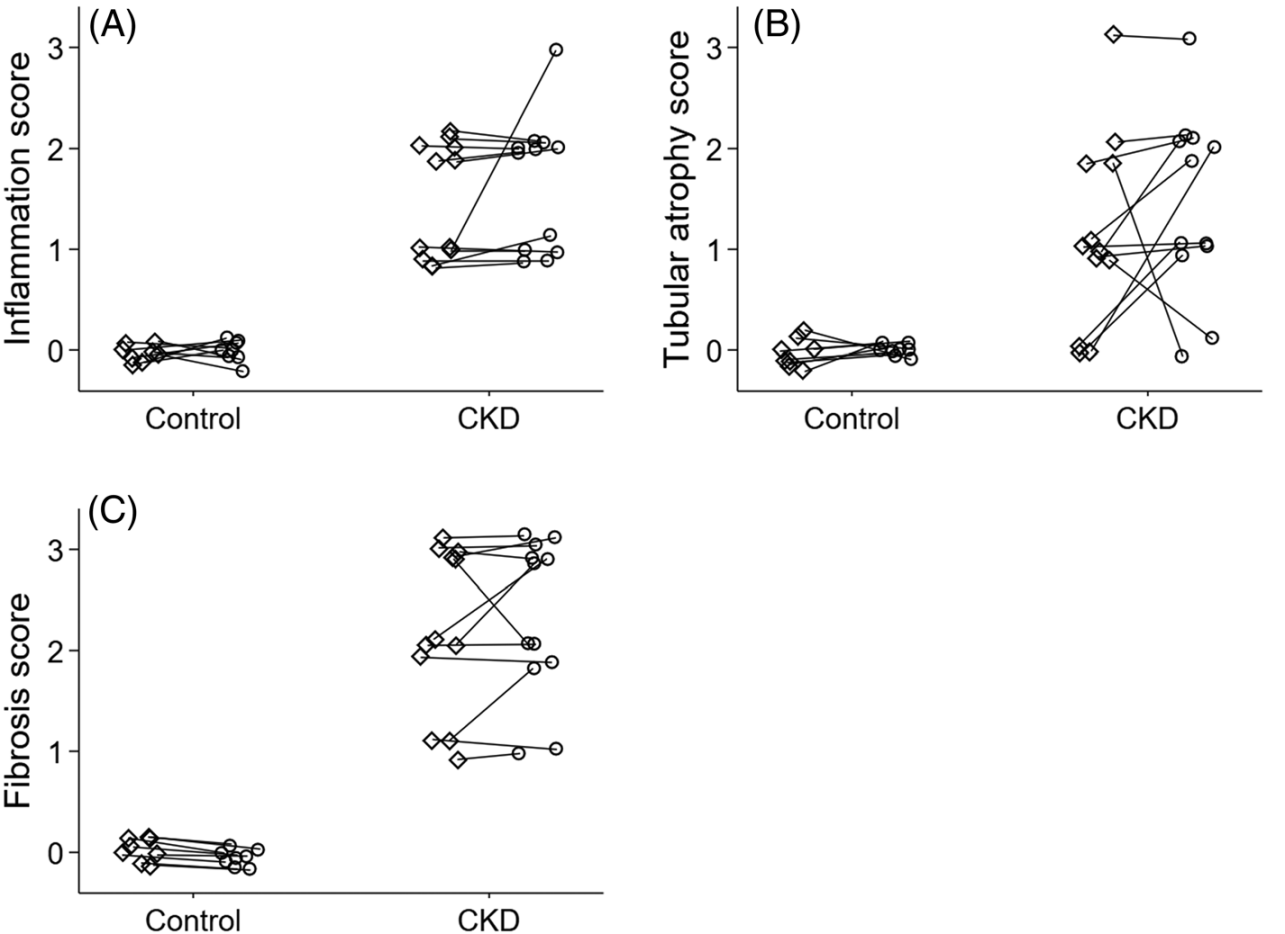
Dot plot of median inflammation (A), tubular atrophy (B), and fibrosis (C) scores of kidneys from cats with chronic kidney disease (n = 12) and from healthy adult control cats (n = 8). Values from the left (open diamond) and right kidney (open circle) of the same individual are connected by solid lines. Histologic scores were recorded as integer values; spherical random noise has been added to the observations in this figure to facilitate visualization of kidneys with overlapping scores

In accordance with study inclusion criteria, control cats had normal renal histologic evaluations with renal scores of zero for inflammation, tubular atrophy, and fibrosis. For CKD cats, median (range) inflammation, tubular atrophy, and fibrosis scores were 2 (1‐3), 1 (0‐3), and 2 (1‐3), respectively (Figure 2).

### 3.5 Association between gene transcript levels and histologic scores of diseased kidneys

**FIGURE 3:**
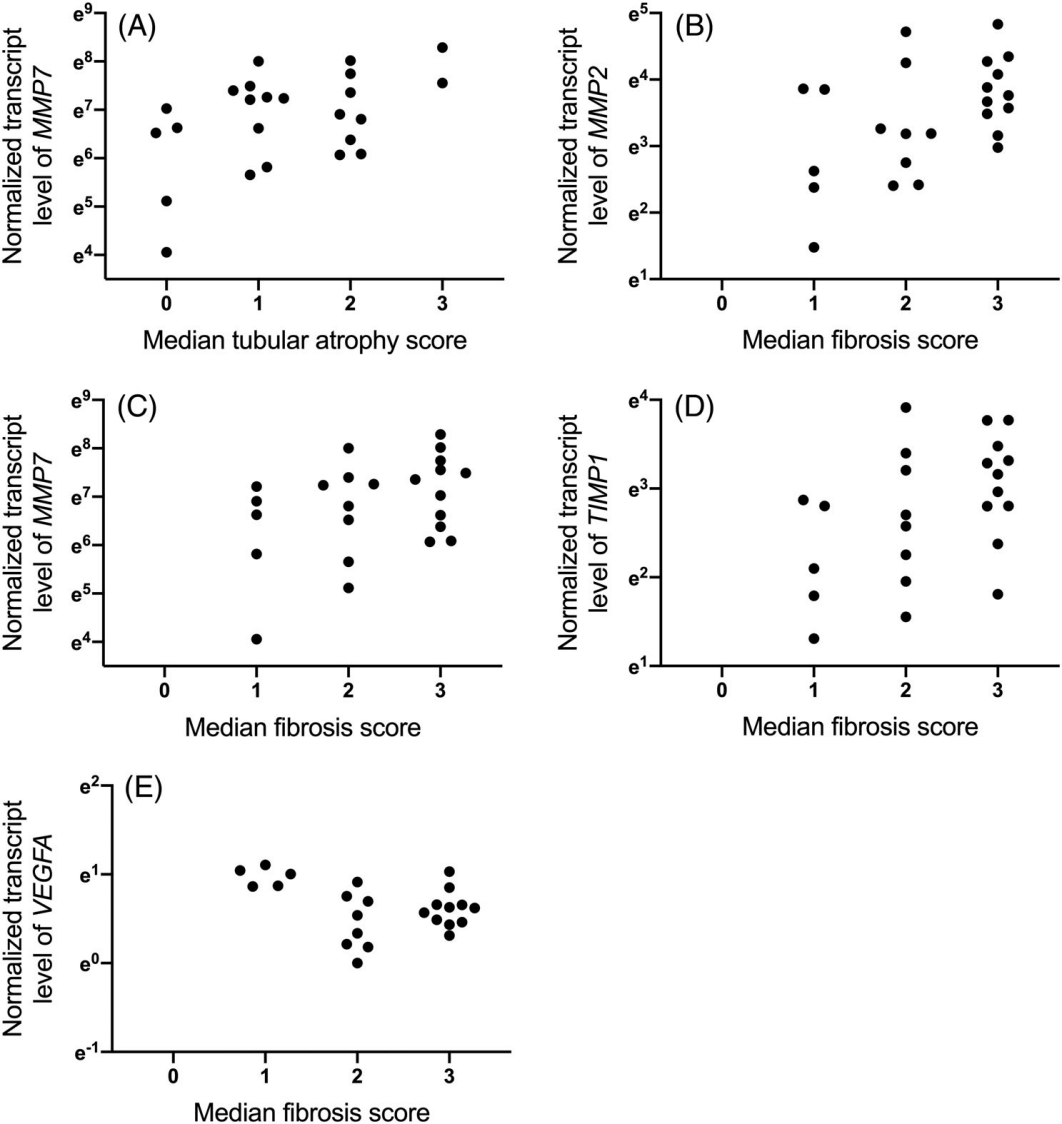
Scatter plot of natural log of normalized gene transcript levels of *MMP7* and tubular atrophy scores (A), *MMP2* and fibrosis scores (B), *MMP7* and fibrosis scores (C), *TIMP1* and fibrosis scores (D), and *VEGFA* and fibrosis scores (E) in tissue samples from each kidney of cats with chronic kidney disease (n = 12)

In the kidneys from the CKD group cats, there was a significant, positive association between transcript levels of *MMP7* and median tubular atrophy scores, and between transcript levels of each of *MMP2*, *MMP7*, and *TIMP1* and median fibrosis scores (Table 4; Figure 3). Transcript levels of *VEGFA* were significantly, negatively associated with median fibrosis scores of affected kidneys.

**TABLE 4:** Association between renal histologic lesion scores for inflammation, tubular atrophy, and fibrosis, and natural log‐transformed gene transcript levels in bilateral renal tissue samples from cats with chronic kidney disease (n = 12) using a linear mixed model

Histologic score	Gene	Coefficient (SE)	*P* value
Inflammation	*HIF1A*	0.49 (0.24)	.05
	*MMP2*	0.15 (0.28)	.61
	*MMP7*	0.42 (0.36)	.25
	*MMP9*	−0.51 (0.39)	.21
	*TGFB1*	0.34 (0.18)	.06
	*TIMP1*	0.18 (0.26)	.51
	*VEGFA*	−0.09 (0.11)	.44
Tubular atrophy	*HIF1A*	0.08 (0.16)	.62
	*MMP2*	0.31 (0.15)	.05
	*MMP7*	0.64 (0.19)	.003
	*MMP9*	0.22 (0.23)	.34
	*TGFB1*	0.16 (0.12)	.18
	*TIMP1*	0.22 (0.15)	.16
	*VEGFA*	0.04 (0.07)	.58
Fibrosis	*HIF1A*	0.06 (0.18)	.75
	*MMP2*	0.67 (0.19)	.002
	*MMP7*	0.71 (0.27)	.02
	*MMP9*	0.66 (0.33)	.06
	*TGFB1*	0.08 (0.14)	.57
	*TIMP1*	0.49 (0.19)	.02
	*VEGFA* ^a^	−0.43 (0.12)	.003

*Note*. Linear mixed model with fixed effect = histologic score; random effect = cat.

Abbreviations: *HIF1A*, gene for hypoxia‐inducible factor‐1α; *MMP2*, gene for matrix metalloproteinase‐2; *MMP7*, gene for matrix metalloproteinase‐7; *MMP9*, gene for matrix metalloproteinase‐9; *RPS7*, gene for ribosomal protein S7; *TGFB1*, gene for transforming growth factor‐β1; *TIMP1*, gene for tissue inhibitor of metalloproteinase‐1; *VEGFA*, gene for vascular endothelial growth factor‐A.

a The coefficient for the *VEGFA* fibrosis model represents the change in ln(*VEGFA* transcript levels) for kidneys with a fibrosis score of 2 (moderate) or 3 (severe) compared to kidneys with a fibrosis score of 1 (mild). The coefficients for all other models represent the change in ln(transcript levels) corresponding to a one‐unit increase in the linear histologic score.

## 4 DISCUSSION

The present study describes upregulation of gene transcription for several fibrosis mediators, including *HIF1A*, *MMP2*, *MMP7*, *MMP9*, *TIMP1*, and *TGFB1*, as well as downregulation of the proangiogenic factor *VEGFA*, in renal tissues from cats with naturally occurring CKD, when compared to those from healthy control cats. In diseased kidneys, gene transcript levels of *MMP2*, *MMP7*, and *TIMP1* were positively associated with worsening histologic lesion scores. These molecular pathways are similarly differentially transcribed in kidneys from cats subjected to transient unilateral renal ischemia as a model of renal fibrosis and CKD.^22^ Collectively, these data strengthen the evidence supporting the role of hypoxia in the intrinsic progression of CKD,^6^, ^7^, ^8^ and suggest specific candidates for biomarkers of tubulointerstitial inflammation and fibrosis in cats.

In tissues from cats with CKD, transcript abundance of *MMP2* and *MMP7* was correlated with worsening degrees of renal fibrosis. Tubulointerstitial fibrosis is highly correlated with functional impairment.^2^, ^3^ Thus, it was not surprising that transcript levels of *MMP7* were also positively associated with sCr in the CKD group in the present study. In human beings, MMP‐7 is recognized as both an important mediator of fibrosis in CKD, as well as a urinary biomarker of renal fibrosis.^17^, ^33^ Our data suggest that MMP‐7 may similarly represent a useful biomarker for this histologic pattern of change in cats; however, whether increased transcription of the *MMP7* gene is accompanied by increased urinary levels of this protein remains to be investigated.

The gelatinase MMP‐2 also appears to play a pivotal role in the progression of interstitial fibrosis.^34^ Serum concentrations of both MMP‐2 and MMP‐9 and their inhibitors TIMP‐1 and TIMP‐2 were increased in children with CKD when compared to age‐matched controls, and these concentrations were elevated in proportion to disease stage.^35^ Conversely, in adult human beings with CKD, serum activity of MMP‐2 was increased, whereas activity of MMP‐9 was decreased relatively to control subjects.^36^ In that report, sCr was directly correlated with MMP‐2 activity and inversely correlated with MMP‐9 activity. Although no significant association between renal transcript levels of *MMP2* and sCr was observed in the present study (*P* = .06), the number of subjects evaluated was small. Also, this study did not evaluate the relationship between renal transcript levels and the corresponding protein levels in serum and urine, which warrants future examination.

Notably, 7 of the cats in our study displayed focal papillary and pelvic urothelial changes consisting of hyperplasia with or without cellular atypia. Studies in human beings document an association between increased urinary activities of MMP‐2 and MMP‐9 and early‐stage bladder carcinoma.^37^ Balkan endemic nephropathy, a familial chronic tubulointerstitial disease characterized by tubulointerstitial nephritis in people with histologic features similar to those of CKD in cats,^1^, ^38^ is associated with the development of papillary transitional cell carcinoma of the renal pelvis, ureter or both.^39^ If the urothelial changes observed in the cats in our study represent preneoplastic lesions, it is possible that these lesions could have contributed to the observed upregulation of *MMP2* and *MMP9*. Nonetheless, an upregulation of these MMPs occurs in cats with experimentally induced ischemic CKD, which lacked atypical urothelial changes on histology.^22^

In a previous study, renal transcription of *HIF1A* was not different between cats with experimentally induced, ischemic CKD and control cats; however, it was positively correlated with worse fibrosis scores in diseased kidneys.^22^ Conversely, in the present study, which evaluated kidneys from cats with more severe histologic and functional derangements than those of the earlier study, a significant difference in transcription abundance of *HIF1A* was observed in renal tissues from cats with CKD as compared to those from normal controls; nonetheless, an association with histologic scores was not found. These data suggest that chronic renal hypoxia might be an ongoing feature of CKD in cats but that *HIF1A* transcription might not be consistently proportional to disease severity. Work performed in cultured renal cells and knockout mice models suggests that activation of HIF‐1 signaling in renal epithelial cells is associated with the development of chronic renal disease by promoting fibrogenesis.^40^, ^41^ Therefore, HIF‐1α signaling might warrant evaluation as a therapeutic target in cats.

In the present study, transcription of *TGFB1* was upregulated in CKD. Two prior veterinary reports document increased urinary TGF‐β1 levels (expressed as urinary TGF‐β1‐to‐creatinine ratio) in cats with naturally occurring CKD, as compared to healthy control cats.^18^, ^21^ Recently, a positive correlation between this ratio and sCr was identified.^18^ We failed to find a similar association in the present study and although TGF‐β1 is a prototypical fibrogenic cytokine, there was no significant association between its transcript levels and the median histologic lesion scores. As regulation of gene expression occurs at the level of both transcription and translation,^42^ it is possible that assessment of transcript levels may not directly reflect levels of TGF‐β1, which may have influenced our results. Additionally, as discussed below, the present study design may have introduced biases regarding sCr values either by utilizing measurements that were not contemporaneous with tissue collection, or that were obtained during a period of acute decompensation.

In addition to documenting increased urinary TGF‐β1‐to‐creatinine ratio in cats with CKD, Habenicht and colleagues noted that cats with CKD had significantly lower urinary VEGF‐to‐creatinine ratio than did normal cats.^18^ In a separate study, urinary VEGF‐to‐creatinine ratio was inversely associated with the development of azotemia after treatment of hyperthyroid cats.^19^ These findings are in accordance with the downregulation of renal transcription of *VEGFA* described in the CKD cats of present study. It has been hypothesized that decreased production of VEGF may promote loss of peritubular capillary density and contribute to progressive renal disease in CKD.^43^ Nevertheless, the role of this growth factor in the pathophysiology of renal disease is complex. Conflicting data from different models and distinct forms of renal disease describe it as deleterious in some disease settings and protective in others.^44^

There are limitations to this study. First, cats with CKD were significantly older and more likely to be sexually altered (via orchiectomy or ovariohysterectomy) than control cats. Although age was not significantly associated with the transcript levels of any gene in our study, the impact of neuter status was not explored. Furthermore, as posttranscriptional regulation of gene expression was not examined, any impact of age or sex in the regulation of gene expression would have been missed. Additionally, cats with CKD were obtained from a population of client‐owned cats, whereas control cats were obtained from feral or purpose‐bred cat populations. Therefore, results may be influenced by differences in dietary or environmental factors, or both. Of relevance, renal tissues were procured after death or euthanasia and a bias toward animals that may have been experiencing relatively rapid disease progression is likely in the CKD group. To this point, an acute exacerbation of renal disease was not able to be distinguished from stable CKD in all cases. It is possible that an overrepresentation of acute‐on‐chronic disease biased our results toward patterns observed in acute kidney injury. As blood and urine sampling was not performed in client‐owned cats, clinicopathologic data from cats with CKD were collected at various time points before euthanasia. Thus, these data may not accurately reflect disease state at the time of collection, either by representing an earlier time point, or by being acquired during a period of decompensation, when the patient may have been more likely to experience prerenal azotemia. To address this, the authors limited the analyses measuring association between gene transcription and sCr to cats for which biochemical data was obtained within 3 months of euthanasia. Nonetheless, the potential impact of these factors must be considered when interpreting the reported correlations between gene transcript levels and sCr. Importantly, the number of individuals examined in the present study was small. Therefore, the study may have been underpowered to detect additional significant associations between gene transcription and biochemical or histologic parameters in the CKD population. Furthermore, the associations noted here may not remain significant if examined in a larger population of cats. Finally, the present study's assessment of the evaluated pathways was limited to the quantification of gene transcript levels using RT‐qPCR. As the expression of the proteins coded by these genes was not quantified, the definitive role of these markers in the pathophysiology of tubulointerstitial fibrosis in cats remains to be clarified.

In conclusion, kidneys from cats with CKD included in the present study showed upregulation of transcription of profibrotic cytokines and growth factors known to be induced by ischemic injury. Additionally, transcript levels of *MMP2*, *MMP7*, and *TIMP1* were positively associated with the severity of histologic scores. Future evaluation of the proteins corresponding to the genes evaluated here in a larger population of cats is warranted to explore their potential as biomarkers of renal fibrosis and as targets for therapeutic intervention in feline CKD.

## CONFLICT OF INTEREST DECLARATION

Dr Bianca Lourenço was the recipient of a Boehringer Ingelheim Postdoctoral Scholarship.

## OFF‐LABEL ANTIMICROBIAL DECLARATION

Authors declare no off‐label use of antimicrobials.

## INSTITUTIONAL ANIMAL CARE AND USE COMMITTEE (IACUC) OR OTHER APPROVAL DECLARATION

The University of Georgia IACUC approved all activities related to this study (Animal Use Protocol A2017 05‐008‐Y3‐A1).

## HUMAN ETHICS APPROVAL DECLARATION

Authors declare human ethics approval was not needed for this study.
